# Transporter-Mediated Subcellular Distribution in the Metabolism and Signaling of Jasmonates

**DOI:** 10.3389/fpls.2019.00390

**Published:** 2019-04-02

**Authors:** Feifei Wang, Guanghui Yu, Pei Liu

**Affiliations:** Department of Ecology, College of Resources and Environmental Sciences, China Agricultural University, Beijing, China

**Keywords:** jasmonates, biosynthesis, metabolism, signaling, jasmonate transporters, subcellular distribution, plasma membrane, nuclear envelope

## Abstract

Jasmonates (jasmonic acid and its relatives) are a group of oxylipin phytohormones that are implicated in the regulation of a range of developmental processes and responses to environmental stimuli in plants. The biosynthesis of JAs occur sequentially in various subcellular compartments including the chloroplasts, peroxisomes and the cytoplasm. The biologically active jasmonoyl-isoleucine (JA-Ile) activates the core JA signaling in the nucleus by binding with its coreceptor, SCF^COI1^-JAZ. Five members of a clade of ATP-binding cassette G (ABCG) transporters of *Arabidopsis thaliana* were identified as the candidates of jasmonate transporters (JATs) in yeast cells. Among these JATs, AtJAT1/AtABCG16, has a dual localization in the plasma membrane and nuclear envelop and mediates the efflux of jasmonic acid (JA) across the plasma membrane and influx of JA-Ile into the nucleus. Genetic, cellular and biochemical analyses have demonstrated that AtJAT1/AtABCG16 is crucial for modulating JA-Ile concentration in the nucleus to orchestrate JA signaling. AtJAT1 could also be involved in modulating the biosynthesis of JA-Ile by regulating the distribution of JA and JA-Ile in the cytoplasm and nucleus, which would contribute to the highly dynamic JA signaling. Furthermore, other JAT members are localized in the plasma membrane and possibly in peroxisomes. Characterization of these JATs will provide further insights into a crucial role of transporter-mediated subcellular distribution in the metabolism and signaling of plant hormones, an emerging theme supported by the identification of increasing number of endomembrane-localized transporters.

## Partition of the Biosynthesis and Signaling of Jasmonates in Various Subcellular Compartments

Jasmonates (jasmonic acid and its relatives, JAs) are a class of oxylipin phytohormones that play important roles in various developmental processes, responses to abiotic and biotic stimuli, in particular immunity against herbivores or pathogen attack (Browse, 2009; Wasternack et al., 2013). JA biosynthesis occurs sequentially in the chloroplasts and peroxisomes, starting from the releasing of α-linolenic acid (18:3) (α-LeA) from galactolipids of chloroplast membranes likely catabolized by a phospholipase1 (PLA_1_). The α-LeA is then oxygenated by 13-lipoxygenase (13-LOX) to form (13S)-hydroperoxy-linolenic acid, which is rearranged and cyclized by allene oxide synthase (AOS) and allene oxide cyclase (AOC) to form 12-oxo-phytodienoic acid (OPDA). OPDA is then translocated to the peroxisomes, where it is converted to JA after reduction by 12-oxophytodienoate reductase (OPR) and three steps of β-oxidation by acyl-CoA-oxidase. JA is then translocated to the cytosol and converted to a number of JA derivatives, among which the biologically active JA conjugate, jasmonoyl-isoleucine (JA-Ile), is formed by jasmonate resistant 1 (JAR1) (Browse, 2009; Fonseca et al., 2009; Gfeller et al., 2010; Song et al., 2011; Wasternack and Hause, 2013). JA-Ile is then bound with its coreceptor SCF^COI1^-JAZ, targeting the jasmonate ZIM domain (JAZ) transcriptional repressors for ubiquitination and degradation. The degradation of JAZ proteins relieves their repression on the activity of the key transcription factors (e.g., MYC2), activating the core JA signaling pathway (Chini et al., 2007; Thines et al., 2007; Browse, 2009; Sheard et al., 2010). Both the COI1 and JAZ proteins are localized in the nucleus and their nuclear localization was not altered in the mutants of the other interacting partner (Chini et al., 2007; Withers et al., 2012). These findings suggest that the assembling of the SCF^COI1^-JAZ-JA-Ile receptor-ligand complex occurs in the nucleus, thereby the nuclear entry of JA-Ile is required.

Besides JA-Ile, JA can also be converted to other derivatives by glycosylation, hydroxylation, methyl esterification and sulfonation, which could be involved in modulating the homeostasis of JAs, cross-talking with other signals and/or could act as signaling molecules. JA conjugates such as sugar (e.g., glucose and gentiobiose)-conjugated JAs are likely storage forms of JAs to regulate the activation/deactivation of JA-Ile mediated core JA signaling pathway by modulating the availability of JA in the cytosol. Furthermore, the hydroxylated JA, (-)-12-hydroxyJA (tuberonic acid, TA), (-)-9,10-dihydroJA, and (-)-11,12-dihydroJA (Yoshihara et al., 1989) may also be involved in regulating the availability of cytosolic JA for the biosynthesis of JA-Ile. In addition, oxidation of JA-Ile mediated by wound-inducible cytochromes P450, CYP94C1, and CYP94B3 are involved in the inactivation of JA-Ile (Heitz et al., 2012). Although the cytoplasmic localization of enzymes catabolizing reactions to produce these JA derivatives was only demonstrated experimentally for JAR1, it is generally accepted that these reactions occur in the cytoplasm. JA carboxy methyl transferase (JMT) catalyzing the formation of methyl-JA (MeJA), a volatile involved in plant-plant communication, is also thought to localize in the cytosol (Seo et al., 2001). In addition, the biosynthesis of *cis*-jasmone (CJ) was proposed to occur in the peroxisomes by decarboxylation of JA and act as the disposal pathway of JA due to its high volatility (Koch et al., 1997), though an alternative origin from *iso*-OPDA has been proposed (Dabrowska and Boland, 2007). CJ is critical for indirect defense by attracting the predators/parasites of herbivores (e.g., seven-spot ladybirds as a kairomone) directly as a semiochemical (Birkett et al., 2000) and indirectly as a signaling molecule (Bruce et al., 2008). Through a signaling pathway distinct from JA-Ile, CJ triggers emitting of a mixture of volatile organic compounds (VOCs) critical for beneficial animal attraction (Birkett et al., 2000; Bruce et al., 2008; Matthes et al., 2010). Although more data remain to be furnished, the biosynthesis and signaling of JAs has been shown to partition in distinct subcellular compartments including chloroplasts, peroxisomes, cytosol and nuclei. Therefore, the distribution of JAs in different subcellular compartments would significantly modulate the metabolism and signaling of these JAs.

## Subcellular and Long Distance Transport of Jasmonates

Information on the intracellular transport of JAs is limited, due to technical difficulties in measuring JAs in different subcellular compartments (Skalicky et al., 2018). The significance of subcellular distribution on the metabolism and signaling of JAs has been revealed by the identification of peroxisomal ATP-binding cassette (ABC) COMATOSE (CTS) transporter. CTS represents the key point for the peroxisomal entry of the substrates of β-oxidation including the key JA precursor, 12-oxophytodienoic acid (OPDA) (Footitt et al., 2007). However, null *cts* alleles do not show the characteristic male sterility of mutants defective in JA biosynthesis or signaling. The *cts* mutants have residual basal levels of JA and can synthesize a modest amount of JA in response to wounding, suggesting that OPDA could also be imported into peroxisomes via passive diffusion or a second importer (Theodoulou et al., 2005). Impaired OPDA import in *cts* mutants leads to elevated levels of this signal molecule in the cytosol, triggering the activation of the distinct OPDA signaling pathway (Dave and Graham, 2012; Maynard et al., 2018). These findings highlight that CTS-mediated distribution of OPDA between the cytosol and peroxisome plays an essential role in regulating the homeostasis and signaling between OPDA and JA.

Besides transport between subcellular compartments, long distance transmission of JAs could play an essential role in triggering wound-induced systemic resistance (WSR) in the distal undamaged leaves. In tomato and tobacco, reciprocal stem-stem grafting between wild type and mutants defective in JA biosynthesis and signaling showed that both the biosynthesis of JA/JA-Ile at the local damaging sites and the capability to perceive JA signal in the distal sites were required, indicating that JAs could act as the mobile wound signal (Li et al., 2002, 2005; Schilmiller and Howe, 2005; Bozorov et al., 2017). Consistently, radiolabeled JAs show that JA (Sato et al., 2009; Bozorov et al., 2017), MeJA (Thorpe et al., 2007), and JA-Ile (Sato et al., 2011) can all translocate from the local damaged to the distal undamaged leaves. Consistent with the observation in the wild tobacco *N. sylvestris* that exogenous radio-labeled JA can translocate from the older leaves to the younger leaves, the long-distance transport of ^11^C-labeled MeJA was via the phloem pathway in *N. tabacum* (Zhang and Baldwin, 1997). Inconsistent results have been reported on the long-distance transport of radiolabeled JA-Ile (Wang et al., 2008; Sato et al., 2011), however, grafting experiments and biochemical studies indicate that JA, but not JA-Ile, is the most likely candidate of the mobile oxylipin signal (Gasperini et al., 2015; Bozorov et al., 2017). In contrary, biochemical studies show that wound-induced JA/JA-Ile in systemic leaves is predominantly derived from the *de novo* synthesis, rather than transported from the local damaged leaves (Matsuura et al., 2012) and the mobile signal moves at a faster speed than that of solute transport along the phloem in *Arabidopsis*. These results cast doubts on whether JAs could act as the transmissible wound signals (Farmer et al., 2003; Windt et al., 2006; Glauser et al., 2009; Koo et al., 2009; Mousavi et al., 2013). However, this debate would be reconciled by postulating that an iterative JA auto-amplification process along the phloem network could rapidly transmit the wound signal(s) to the distal leaves (Truman et al., 2007). However, this self-reinforcing mode of JA signal relay requires rapid cell-cell transport of JA (Schilmiller and Howe, 2005; Truman et al., 2007). Therefore, transporters mediating active transport of JAs has been long predicted. Furthermore, inhibitors of membrane transporters inhibited the loading and accordingly the long-distance transport of the radio-labeled MeJA (and the converted ^11^C-JA) (Thorpe et al., 2007), supporting that transporter(s) may mediate the active loading and long-distance transport of JAs in the phloem.

## Transporters of Jasmonates

Similar to other phytohormones including salicylic acid (SA), abscisic acid (ABA), indole acetic acid (IAA), and gibberellic acid (GA), JA is also a weak acid with a pK_a_ of ∼4.5 that will be trapped (in the form of JA^-^) in the relatively alkaline cytosol (pH, 7.2) rather than in the apoplasm (pH, 5.5) (Robert and Friml, 2009; Kang et al., 2010). It has been long postulated that transporters are essential to mediate cellular efflux of these phytohormones into the apoplasm in the source cells. In addition, importers mediating the influx of these molecules from the apoplasm into the cells would facilitate the uptake of the phytohormones in the target cells, thereby promoting cell-cell transport. A number of plasma-membrane localized transporters have been identified for plant hormones, in particular for IAA (Park et al., 2017). The active transport of most of these plant hormones implicates the ATP-binding cassette (ABC), particularly the ABCG family, transporters and Nitrate transporter1/peptide transporters (NPFs). While most of the ABC transporters exhibited relatively higher substrate specificity of transport, the NPF proteins usually have broad substrates (Park et al., 2017).

Several member of NPFs were identified in a yeast-two-hybrid assay that detected JA-Ile-dependent binding of COI1 and JAZ3 as the candidates of jasmonate transporters (JATs). Using LC-MS/MS, a JA-Ile transport activity has been demonstrated for NPF4.1/AIT3 in yeast cells (Chiba et al., 2015). Another NFP member NPF2.10/GTR1 showed activity for the uptake of JA-Ile and JA in Xenopus oocytes (Saito et al., 2015). Their role in JA transport, however, remains to be clarified *in planta*. Furthermore, NPF4.1/AIT3 also showed transport activity for ABA and GAs in yeast cells (Chiba et al., 2015), while NPF2.10/GTR1 showed transport activity for GAs, NO3^-^ and glucosinolates (Saito et al., 2015). Although the translocation of JA/JA-Ile from local wounded leaves to distal undamaged leaves was impaired in *GTR1* loss-of-function mutant, JA-Ile mediated core JA signaling was increased (Ishimaru et al., 2017) and exogenous GA3, but not JA, recovered male fertility to *gtr1* mutant (Saito et al., 2015). These results indicate that multiple-functional NPF4.1/AIT3 and NPF2.10/GTR1 with JA/JA-Ile transport activity may primarily involve in the interplays between JA and other signals (e.g., GA).

**FIGURE 1:**
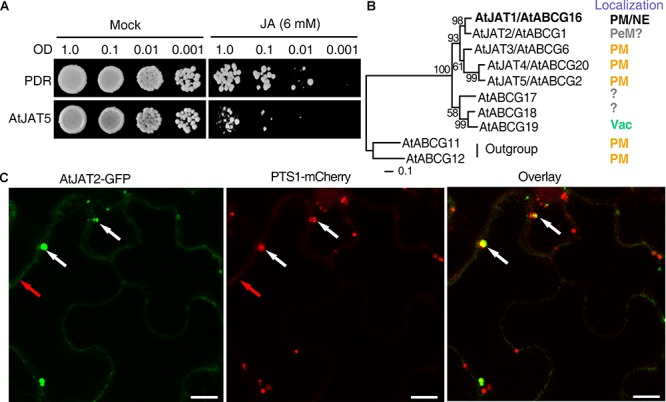
Identification of jasmonate transporters (JATs) in yeast cells and the diverse subcellular localization of JAT family members. **(A)** Exogenous JA (6 mM) inhibits the growth of yeast cells. Yeast cells were diluted (OD_600_= 1.0, 0.1, 0.01, and 0.001) and grown on SD-URA plate (Mock) or SD-URA supplemented with exogenous JA for 5–7 days. As illustrated for AtJAT5, putative jasmonate transporters (JATs) were identified by altered sensitivity to exogenous JA when the candidates were heterogeneously expressed in yeast cells. **(B)** Phylogenic relationship of 5 ABCG members of JAT family and their subcellular localization (PM, plasma membrane; NE, nuclear envelope; PeM, peroxisomal membrane; Vac, Vacuole). The question marks indicate the subcellular localizations that are tentatively inferred (e.g., AtJAT2) or unkown. AtJAT1 that has been characterized as a jasmonate transporter was indicated by bold letters. **(C)** The AtJAT2-GFP signal was colocalized with PTS1-mCherry/RFP (the peroxisomal marker) signals predominantly in the peroxisomes (indicated by white arrows), although weak signal was also detected in the PM (indicated by red arrows) in the epidermal cells of tobacco. The confocal microscopy analyses were performed 2 days after coinfiltration of *Agrobacterium tumefaciens* strains EHA105 carrying AtJAT2-GFP and PTS1-RFP constructs. Scale bars, 10 μm.

Exogenous JA can inhibit the growth of yeast cells, thus we exploited this system to screen JATs in yeast cells (Figure 1A). Three genes encoding a clade of half-molecule ABCG proteins with 5 members (AtABCG1, AtABCG2, AtABCG6, AtABCG16, and AtABCG20) were induced by exogenous JA treatment in stamens (Mandaokar et al., 2006), and thus are the most likely candidates of JATs (Figure 1B). In addition, AtABCG2, 6, and 20 function redundantly in the synthesis of effective suberin barriers of seed coats and roots and the exine of pollen coat (Yadav et al., 2014). However, the preferential expression of these ABCGs in the vascular tissues indicates that they are unlikely involved directly in the synthesis of suberin. The *atabcg1abcg16abcg20* triple mutant exhibited male sterility (Yadav et al., 2014), characteristics of mutants in JA biosynthesis and signaling, providing genetic evidence to support an involvement of these ABCGs in JA signaling. Yeast cells expressing AtABCG16 exhibited enhanced sensitivity to exogenous JA and increased cellular JA retention, indicating that AtABCG16 is a jasmonate transporter (named as AtJAT1) (Li et al., 2017). More interestingly, an additional localization of AtJAT1/ABCG16-GFP in, besides plasma membrane (PM), the nuclear envelope (NE) in transgenic *Arabidopsis* plant has been revealed. Utilizing cultured suspension cells and isolated nuclei, AtJAT1/AtABCG16 was shown to mediate the cellular export of JA and nuclear import of JA-Ile in a high-affinity mode (Li et al., 2017). The *atjat1* mutant show significantly compromise in JA-Ile-mediated core JA signaling as evident by expression of JA-responsive genes, JA-Ile induced degradation of JAZ1 protein and resistance to the necrotrophic fungal pathogen, *B. cinerea*. Significantly, *atjat1; atjar1* double mutant, but not *atjat1* or *atjar1* mutant, exhibited characteristic male sterility, which was rescued by exogenous JA. A cooperation between AtJAT1 and AtJAR1, which catabolizes the formation of JA-Ile, in regulating JA-Ile-mediated core JA signaling provide robust genetic evidence to support the finding that AtJAT1 mediates nuclear import of JA-Ile.

Semi-quantitative electron microscopic autoradiography using the *atjar1* mutant cells, in which the forming of the biological active JA-Ile is largely depleted (Suza and Staswick, 2008), showed that JA-Ile, but not JA enter the nuclei. Consistent with a role of ATJAT1 in nuclear import of JA-Ile, JA-Ile in the nucleus was remarkably decreased (Li et al., 2017). AtJAT1-mediated nuclear influx of JA-Ile would ensure cells to accumulate a critical nuclear JA-Ile concentration for activating JA signaling rapidly and substantially. In animals, nuclear receptors are activated by binding with their cognate small-molecule hormonal ligands and function as transcription factors to bind directly with the hormone response elements (HREs) in the promoters of their target genes. The binding of nuclear receptors can occur in either the cytoplasm (type I nuclear receptors e.g., steroid receptors) or the nucleus (type II nuclear receptors e.g., triiodothyronine receptors) (Tata, 2002; Puzianowska-Kuznicka et al., 2013). Although plants do not have canonical nuclear hormone receptors, the receptors for auxin, JA, GA, and ABA are nucleus-localized and these receptors are proposed to bind with their hormonal ligands in the nucleus (Santner and Estelle, 2009; Lumba et al., 2010). Contrary to the view that small molecules diffuse passively through the nuclear pore complex (NPC) (Terry et al., 2007; Meier and Brkljacic, 2010), transport across the nuclear envelope (NE) is mediated by Na/Ca exchanger for Ca^2+^ in cultured animal cells (Wu et al., 2009; Galva et al., 2012) and by P-glycoprotein (P-gp) transporter for small-molecule medicines in multiple drug resistant tumor cells (Szaflarski et al., 2013). The identification of AtJAT1 shows that JA-Ile is actively transported into the nucleus rather than diffused passively through NPC. Removal of JA-Ile from the cytosol will consequently effect the equilibrium between JA and JA-Ile (Woldemariam et al., 2012) or hydroxylation of JA-Ile (Koo et al., 2011). Moreover, AtJAT1-mediated cellular efflux of cytosolic JA and nuclear entry of JA-Ile would significantly impact the metabolism of JA occurred in the cytoplasm, in particular the conjugation of JA with Ile (Staswick et al., 2002). Thus, this AtJAT1-modulated distribution of JA-Ile between nucleus and cytoplasm would play a significant role for the dynamics of JA biosynthesis, metabolism and signaling. The mechanism of the high but distinct substrate specificity of AtJAT1 in mediating the nuclear import of JA-Ile (but not JA) and cellular export of JA (but not JA-Ile) remains to be addressed. To execute their functions in substrate transport, half molecule ABCG proteins interact with their self or other ABCG partners to form homodimers and/or heterodimers, which could determine the subcellular localization and substrate specificity of these transporters (McFarlane et al., 2010; Le Hir et al., 2013). Therefore, the substrate-specificity of AtJAT1 might be established by its interacting partners. More remarkably, how AtJAT1 mediates JA-Ile across NE with double-membranes and whether there is basal diffusion of JA/JA-Ile across the PM/NE await to be addressed in future studies.

## Future Perspectives

**FIGURE 2:**
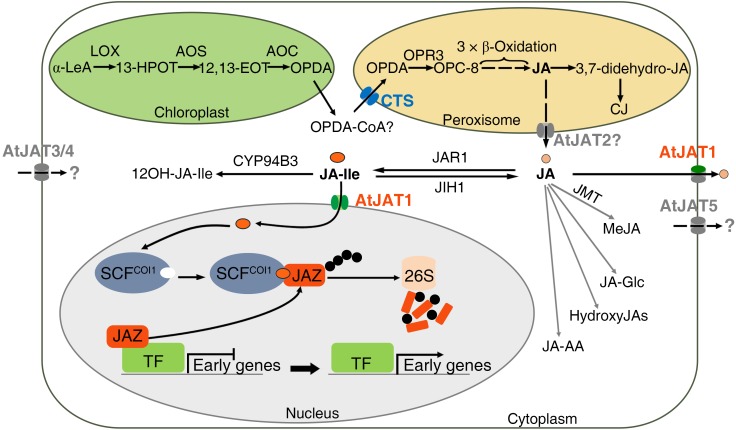
Diverse subcellular localization of JAT family members and their roles in modulating the metabolism and signaling of jasmonates. Schematic model showing transporter-mediated subcellular distribution in modulating the biosynthesis, metabolism and signaling of JAs. The biosynthesis, metabolism and signaling occur sequentially in the chloroplast, peroxisome, cytoplasm and nucleus. CTS is localized in the peroxisome and involved in the peroxisomal entry of the key precursor, OPDA, which is produced in the chloroplast by the oxygenation of α-LeA. The CTS-modulated distribution of OPDA between the cytoplasm and the peroxisome significantly impacts the biosynthesis of JA and accordingly JA and OPDA signaling. AtJAT1 shows dual localizations in the nuclear envelope and plasma membrane and mediates the influx of JA-Ile into the nucleus and efflux of JA from the cytoplasm to the apoplast, accordingly modulating the subcellular distribution of JA-Ile and JA in the nucleus and cytoplasm. Furthermore, AtJAT2 is likely localized in the peroxisomes, which may be involved in the peroxisomal export of JA. While AtJAT3/4/5 are all localized in the PM, AtJAT3/4 may mediate the import of JAs and AtJAT5 may mediate the export of JAs across the PM. The AtJAT2 and AtJAT3/4/5 transporters were indicated in gray since the activity and directionality in jasmonate transport of these transporters remain to be determined. Characterization of these JATs in the future studies will provide further insights in a crucial role of transporter-mediated subcellular distribution in modulating the biosynthesis, metabolism and signaling of JAs.

Due to the PM- and NE-dual localization, AtJAT1 is outstanding among the various phytohormone transporters characterized so far, the majority of which are PM localized (Park et al., 2017). However, dual localizations at the ER and PM of the short PIN (PIN-FORMED) permease-like auxin transporters, which lack the extensive hydrophilic loop separating two transmembrane domains, have also been revealed in *Arabidopsis* epidermal and root hair cells, as well as in tobacco BY-2 cells. These ER-localized PINs presumably regulate auxin homeostasis by pumping auxin into (PIN5) or out (PIN8) of the ER lumen or hypothetically from the ER lumen into the nucleus (PIN6 and PIN8) (Mravec et al., 2009; Bender et al., 2013; Viaene et al., 2013; Park et al., 2017; Skalicky et al., 2018). The identification of AtJAT1 further supports a common theme that the transporter-modulated subcellular partition plays an essential role in modulating the signaling of plant hormones. Since the receptors of IAA, ABA and GA are also nucleus-localized, it is intriguing to know whether these hormonal ligands enter the nucleus, and if so, whether nuclear importers are involved. Direct evaluation of the substrate specificity of hormonal transporters by the transport of radio-labeled chemicals still remains challenging due to the cost and labor to test diverse radiolabeled potential transport substrates. Besides AtJAT1/AtABCG16, the other 4 members (AtABCG1, AtABCG6, AtABCG20, and ABCG2) in the same monophyletic clade may also mediate the transport of jasmonates. Supporting this notion, yeast cells expressing AtABCG2/AtJAT5 showed increased sensitivity to 6 mM exogenous JA (Figure 1A). When transiently expressed in the epidermal cells of tobacco (*N. benthamiana*), AtABCG6/AtJAT3-, AtABCG20/AtJAT4-, and AtABCG2/AtJAT5-GFP signal was previously detected in the PM (Yadav et al., 2014). However, when carefully examined, AtABCG1/AtJAT2-GFP signal was principally detected in the peroxisomes, though weak signal was also observed in the PM when transiently expressed in tobacco epidermal cells (Figure 1C). The localization of AtABCG1/AtJAT2-GFP signal in peroxisomes was later confirmed in the root cells of stable transgenic *Arabidopsis* plants (Manuscript in preparation). Although genetic and biochemical data await to be implemented, we propose that the peroxisome-localized AtJAT2/AtABCG1 might mediate the peroxisomal export of JA (Figure 2). The three PM-localized JATs (AtJAT3/4/5) are among the most likely candidates of JATs. Further evidence supports that AtJAT3/4 mediate the cellular import of JAs and AtJAT5 mediates the cellular export of JAs (unpublished data). The identification of PM-localized JATs will provide an opportunity to address whether JAs act as the mobile signal(s) in systemic wound response (Nguyen et al., 2017). Furthermore, JAT1 could form heterodimers with one of these three ABCGs in mediating cellular export of JA across the PM. As the only JAT that is present in the NE, AtJAT1 may thus form homodimers in mediating nuclear import of JA-Ile. This speculation provides an explanation for the distinct transport properties (substrate specificity and affinity) of AtJAT1 in the PM and NE. The interactions among these PM-localized AtJATs would be important to reveal the possible mechanism to determine substrate specificity of these transporters. Many chemicals, in particular plant secondary metabolites, use the vacuole as the sites for cellular storage, which play an important role in regulating the homeostasis of these chemicals (de Brito Francisco and Martinoia, 2018). A vacuolar auxin transporter facilitator, WAT1, has been identified in *Arabidopsis* and is required for auxin homeostasis and signaling (Ranocha et al., 2013). In the most closely related clade of the JAT family, there are 3 ABCG members (ABCG17, ABCG18, and AtABCG19) (Figure 1B), in which AtABCG19 has been shown to localize in the vacuole and involve in kanamycin resistance (Mentewab and Stewart, 2005). Hence, it is intriguing to know whether these ABCGs also have a transport activity for JAs. In addition, whether there is jasmonate transporter(s) in other transporter family or families still remains an open question. The heterologous system could also be valuable in screening, besides these JATs, such jasmonate transporter(s) in yeast cells. In summary, we propose that the coordinated actions of CTS, AtJAT1 and other uncharacterized JATs play crucial role in modulating the subcellular distribution of OPDA and JAs and accordingly connecting and orchestrating JA biosynthesis, metabolism and signaling partitioned in diverse cellular compartments (Figure 2). The identification of JATs localized in PM and endomembrane of various organelles will provide a unique opportunity to explore the roles of transporter-mediated subcellular partition in modulating the biosynthesis, metabolism and signaling of plant hormones.

## Data Availability

The datasets generated for this study are available on request to the corresponding author.

## Author Contributions

FW collected information on transporters of plant hormones, prepared the figures. GY was involved in preparing the figure. PL conceived the idea and wrote this perspective and collected all other information on jasmonate transporters. All the authors participated in writing and approved the manuscript for publication.

## Conflict of Interest Statement

The authors declare that the research was conducted in the absence of any commercial or financial relationships that could be construed as a potential conflict of interest.
